# The SCOT-HEART Trial. What we observed and what we learned

**DOI:** 10.1016/j.jcct.2019.01.006

**Published:** 2019

**Authors:** Philip D. Adamson, David E. Newby

**Affiliations:** a British Heart Foundation Centre for Cardiovascular Science, University of Edinburgh, Edinburgh, UK; b Christchurch Heart Institute, University of Otago, Christchurch, New Zealand

## 1 Introduction

The diagnosis of suspected stable angina has come a long way since the association between chest pain and coronary artery stenosis was first recognized more than 200 years ago.^1^ Nevertheless, the assessment of exertional symptoms remains a common challenge experienced by cardiologists everywhere. The presenting complaint is frequently atypical in nature, and clinicians are faced with the joint task of avoiding unnecessary investigations whilst also ensuring the safe and efficient identification of those individuals with underlying coronary heart disease. Non-invasive testing strategies have traditionally been dominated by functional assessment of inducible ischemia and have developed over time from the exercise electrocardiogram to myocardial perfusion imaging with single photon emission computed tomography, stress echocardiography, positron emission tomography and magnetic resonance imaging. In each case, there is an apparent association between abnormal test results and both the detection of obstructive coronary artery disease on invasive angiography and the increased risk of adverse cardiovascular events.^2^, ^3^, ^4^, ^5^ However, the prognostic value of abnormal results remains inferior to that provided by anatomical evaluation of the coronary arteries,^6^^,^^7^ and the use of these tests has not been demonstrated to improve clinical outcomes within the context of randomized controlled trials (RCTs).

It is in this context that studies of coronary computed tomography angiography (CCTA) have generated widespread interest within the cardiology community. Initial reports described the diagnostic accuracy of CCTA and highlighted the exquisite sensitivity of this modality.^8^^,^^9^ Subsequently several RCTs have been conducted to provide evidence regarding the relative clinical advantages of anatomic assessment with CT compared with functional testing. Recently, we have reported the 5-year outcomes of the Scottish Computed Tomography of the HEART (SCOT-HEART) trial wherein we identified an important reduction in the composite endpoint of coronary heart disease death or non-fatal myocardial infarction amongst participants randomized to the CCTA intervention.^10^ Although these findings have generally been welcomed by the cardiology community,^11^ understandable questions remain concerning the exact mechanisms by which such benefits were achieved. In examining this uncertainty, we have focussed on several considerations: the importance of trial design, the plausibility of the magnitude of treatment effects, and the consistency of our results within the existing evidence base.

### 1.1 The design of the SCOT-HEART trial

The design of the SCOT-HEART trial has previously been described in detail.^10^^,^^12^^,^^13^ Nevertheless, it warrants review here as it has important implications with regards to the interpretation and clinical application of our results. A pragmatic approach to recruitment was adopted to ensure broad clinical relevance with enrolment open to patients aged 18–75 years who had been referred by a primary-care physician to a dedicated cardiology clinic for patients with suspected stable angina due to coronary heart disease (CHD). Exclusion criteria were kept to a minimum and were predominantly related to suspected acute coronary syndrome or inability to undergo CT scanning (typically due to advanced renal impairment). Individuals with an established history of coronary heart disease remained eligible providing they had not experienced an acute coronary syndrome in the previous 3 months.

All patients underwent routine clinical assessment with 85% of patients in both study groups proceeding to symptom-limited exercise electrocardiography. Symptoms (typical, atypical, or non-anginal chest pain according to the National Institute for Health and Care Excellence [NICE] definition^14^), clinical diagnosis, further planned investigations, and initial treatment strategy were documented at the end of the clinic attendance. Prior to randomisation, clinicians were prompted to categorize the likelihood of the diagnosis of coronary heart disease and angina due to coronary heart disease, and document the subsequent diagnostic strategy including the need for downstream functional imaging, or invasive coronary angiography.

The primary diagnostic endpoint of the study was the diagnostic certainty of patients with angina pectoris secondary to coronary heart disease at 6 weeks. At this juncture, the treating cardiologist was prompted to review their patients’ diagnosis and management plan in view of all available information including the CCTA report (CCTA intervention arm) or the cardiovascular risk score (standard care arm). Clinicians were requested to document any changes to their diagnosis, requirement for additional investigations, or management strategy (medical therapy or coronary revascularization). It is this documentation of management changes that has allowed us to begin exploring some of the plausible mechanisms that might explain the treatment effect observed.

In addition to these immediate impacts, there was a pre-specified principal 5-year outcome comprising a composite of coronary heart disease death or non-fatal myocardial infarction.^13^ In keeping with the pragmatic design of the trial, patients were not required to attend study-related follow-up visits and all clinical events were identified by using the patient-unique Community Health Index (CHI) number to enable linkage to routine electronic health data from the Information and Statistics Division of the National Health Service (NHS) Scotland. As this system is in place nationwide, it ensures complete capture of mortality and hospitalization records, and indeed only 66 patients (1.6% of the total study cohort) emigrated from Scotland during the first 5 years of follow-up.^10^ This approach has previously been demonstrated to perform comparably to more traditional diagnostic adjudication within the West of Scotland Coronary Prevention Study (WOSCOPS).^15^

### 1.2 CCTA and the diagnosis of coronary heart disease

After recruitment, 4146 patients (mean age 57.1 ± 9.7 years, 44% women) were randomly assigned (1:1) to standard care plus coronary calcium score and CCTA (n = 2073), or to standard care alone (n = 2073) with CCTA scans performed using 64 or 320 detector row scanners across three imaging sites.^14^ Amongst those assigned to CCTA, 295 defaulted or did not complete their scan whilst 672 (38%) and 452 (25%) of the remainder had CT evidence of non-obstructive or obstructive CHD respectively. Compared with standard care, CCTA increased diagnostic certainty (relative risk [RR] 2.56, 95% confidence interval [CI] 2.33 to 2.79) and the frequency (RR 1.09, 95% CI 1.02 to 1.17) of a diagnosis of coronary heart disease at 6 weeks. The diagnosis of coronary heart disease at 6 weeks was changed in 27% of those assigned to CCTA compared with 1% of participants assigned to standard care.

### 1.3 CCTA and downstream investigations

The above diagnostic changes were associated with changes in planned investigations for 1 in 6 patients assigned to CCTA including the initial cancellation of 121 functional tests and 29 invasive coronary angiograms, and the initiation of invasive coronary angiography in 94 participants. These changes were mainly the result of the exclusion or identification of obstructive coronary heart disease. Interestingly, although overall there was no difference between groups with regards to the number of invasive coronary angiographic procedures performed during follow-up (491 [23.6%] *versus* 502 [24.2%]; hazard ratio (HR) 1.00 (95% CI 0.88 to 1.13), invasive angiography was less likely to demonstrate normal coronary arteries and more likely to show obstructive coronary artery disease in patients assigned to CCTA.^16^ Furthermore, post-hoc analysis demonstrates a reduced rate of angiography after the first year amongst the CCTA arm of the trial (HR 0.70, 95% CI 0.52 to 0.95).^10^

### 1.4 CCTA and treatment change

Unsurprisingly, changes in diagnostic decision-making were associated with changes in subsequent recommendations for medical therapies and revascularisation procedures. It warrants repeating that attending clinicians were actively prompted to review their treatment decisions in the light of the newly available CCTA results. In contrast, patient management in the standard care group was prompted by existing estimates of cardiovascular risk and use of further non-invasive stress imaging at the discretion of the attending clinician. Correspondingly, nearly 1 in 4 patients in the CCTA group had their prescribed treatment altered at 6 weeks compared with only 1 in 20 (5%) of those receiving standard care alone. Antiplatelet therapy fell from 48% (baseline) to 41% (at 1 year) in the standard of care arm whilst it increased from 49% (baseline) to 52% (at 1 year) in the CCTA group. In contrast, prescriptions for statins increased in both groups (standard care: 43%–50%; CCTA: 44%–59%) but this was greater in the CCTA group (p < 0.001). The greater use of evidence based therapies amongst patients with abnormal findings on CCTA compared with functional testing is consistent with prior reports.^17^ Importantly, prescribing differences regarding preventative therapies were sustained over the 5 years of follow up and these treatments were selectively prescribed to patients who had coronary heart disease documented on the CCTA despite comparable 10-year cardiovascular risk scores. It should also be remembered that the overall rates of prescriptions of preventative therapies do not account for how treatments were changed within the treatment groups, especially those undergoing CCTA, where both cessation and initiation of therapies occur.

Although there was no overall difference in the frequency of coronary revascularization over 5 years (279 *versus* 267, HR 1.07, 95% CI 0.91 to 1.27), the pattern of revascularization does appear to differ between the treatment groups. Specifically, during the first year after randomization, more patients in the CCTA group underwent coronary revascularization (246 *versus* 208, HR 1.21, 95% CI 1.01 to 1.46; p = 0.042), whereas after one year, the rate of coronary revascularization was reduced (33 versus 59, HR 0.59, 95% CI 0.38 to 0.90; p = 0.015).

### 1.5 CCTA and clinical outcomes

At the time of the initial publication of the trial results, the use of CCTA appeared to offer improvements in the composite long-term endpoint of coronary heart disease death or non-fatal myocardial infarction, albeit without reaching the threshold for conventional statistical significance (p = 0.053).^12^ By 5 years, there was a clear reduction in this endpoint amongst those patients who underwent CCTA compared with standard care alone (48 [2.3%] versus 81 [3.9%]; HR 0.59, 95% CI 0.41 to 0.84; p = 0.004). This was primarily driven by a reduction in non-fatal myocardial infarction and, as with other trials, we did not identify any decrease in all-cause mortality. Interestingly, although the 5-year event rates were higher in patients with a prior history of coronary heart disease, similar relative reductions in fatal and non-fatal myocardial infarction were evident in those with and without pre-existing coronary disease.

### 1.6 Time course of benefit and case ascertainment bias

We have previously described an apparent overlap in event curves during the first 50 days after randomization (the median time to change in prescribed therapies amongst the CCTA arm) with the improvement in outcomes associated with CCTA only beginning after that timepoint.^16^ This finding offers helpful insight into the mechanisms responsible for the observed effect size as being predominantly related to changes in preventative therapies, and provides clear evidence that event rates were similar in the two trial arms until downstream treatment changes had been implemented. Furthermore, as the CCTA scans were performed a median of 13 days after randomisation, the lack of an earlier separation of these curves would tend to undermine the proposition that case ascertainment bias is in large part responsible for our findings.

Given that SCOT-HEART was an open trial and that CCTA increased the diagnosis of coronary heart disease, one would anticipate that CCTA would increase the diagnosis of subsequent myocardial infarction and potentially hinder our ability to see a beneficial effect on this outcome. We also demonstrated that CCTA prompted more coronary revascularisations in the first year but beyond one year, rates were markedly reduced. This is in keeping with the CCTA identifying patients at risk early on and leading to the initiation of more appropriate preventative therapies and revascularisation. Patients in the standard of care arm had unrecognised disease which led to the accrual of later events and the need for more downstream coronary revascularisations. Thus, there was an inversion in rates of coronary revascularisations after one year and it is hard to explain how ascertainment bias could lead to such a biphasic change in revascularisation rates.

### 1.7 Plausibility of the magnitude of treatment effect

In determining the planned recruitment for the SCOT-HEART trial, we used historical data from 20 years ago that reported a 13.1% rate of coronary heart disease death or non-fatal myocardial infarction and anticipated a 2.8% *absolute* risk reduction amongst the CCTA group (relative risk 0.81, 95% CI 0.69 to 0.95).^13^^,^^18^ In keeping with many modern cardiovascular trials, the observed number of events was substantially less than this at 3.1% overall with a 1.6% difference between the treatment arms. Consequently, the observed *relative* risk reduction was more than double the magnitude we expected. Nevertheless, these relative risk point estimates have substantially overlapping confidence intervals.

The benefits of preventative therapy trials are well described. However, it should be remembered that many trials (especially primary prevention trials) treated a broad population of patients at risk of cardiovascular disease. Most of these trial participants did not have cardiovascular disease at either study entry or completion. As such, they had no chance of benefiting from the intervention and effect size estimates are diluted and underplay the impact of the trial intervention for those with actual disease. Enriching a population, or better still, identifying a population with the disease before treatment initiation, will potentially lead to greater benefits. Indeed, in the JUPITER trial, the risk stratification step of an elevated high-sensitive c-reactive protein, enabled the identification of a high-risk population who received a more marked benefit from rosuvastatin (hazard ratio, 0.56 (95% confidence interval, 0.46 to 0.69), P < 0.001) than has been seen in other prevention trials.^19^

Because of the broad inclusion criteria, the trial population of SCOT-HEART had a spectrum of at risk participants. Although patients had stable chest pain, many participants had recent onset chest pain, a particularly high-risk group that represents a form of unstable angina.^20^ As such, larger relative benefits will be achieved by aspirin therapy, and early coronary revascularization will reduce the risk of myocardial infarction (Table 1). This likely underlies the early divergence of the event curves seen after 50 days.

Finally, changes in diagnosis can have marked beneficial effects: the right patient gets the right treatment. Indeed, the introduction of sensitive troponin assays to better diagnose acute myocardial infarction has been associated with marked reductions in the risk of cardiovascular death and recurrent myocardial infarction (odds ratio 0.42).^21^

### 1.8 Mechanism of treatment effect

**Fig. 1:**
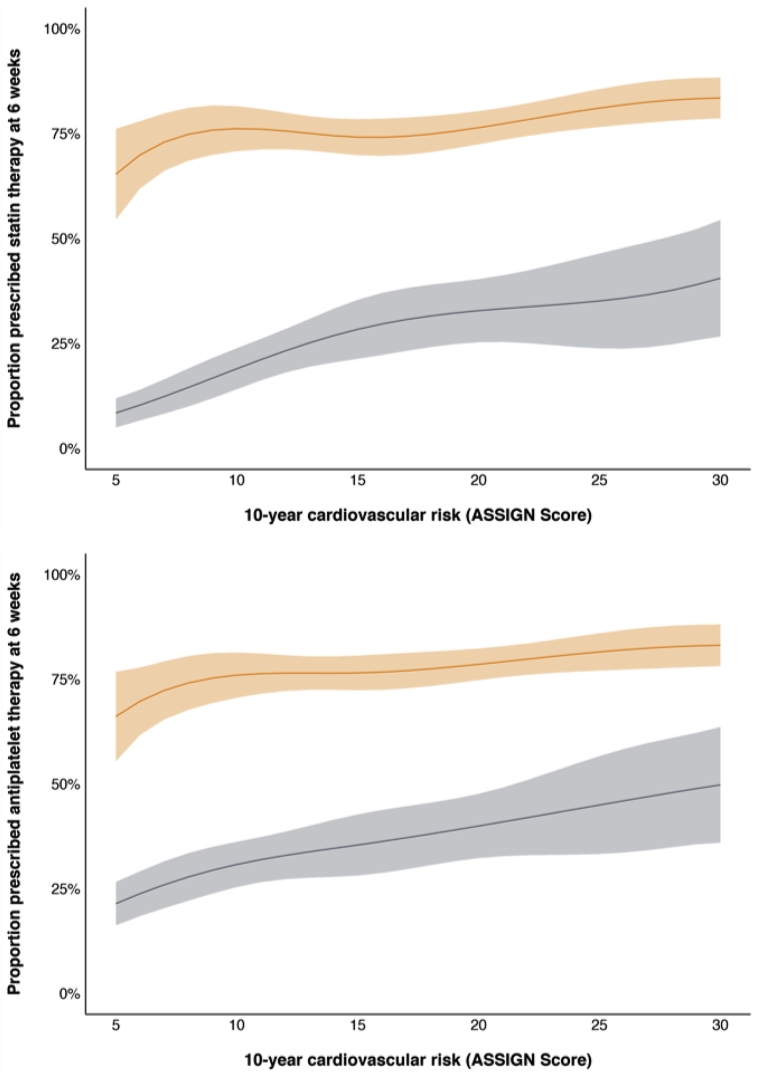
Frequency of prescribing for antiplatelet (top) and statin (bottom) therapy at 6 weeks in patients with (orange) and without (grey) coronary artery disease on computed tomography coronary angiography across a range of 10-year cardiovascular risk as determined from the ASSIGN score. The lines and corresponding shaded areas represent the prescribing estimates and 95% confidence interval derived from a regression model. The ASSIGN is a risk model derived and validated within Scotland for the determination of cardiovascular risk in patients without known coronary heart disease (For interpretation of the references to colour in this figure legend, the reader is referred to the web version of this article.).

It is clear that CCTA does not reduce events in isolation, rather it is a series of responses to the anatomical information CT provides that governs the benefits we observed. Some of these responses have already been described, and include increased prescribing of evidence-based medications and an early increase in coronary revascularization. Others are more difficult to quantify but may include increased disease awareness amongst patients prompting greater efforts to achieve healthy lifestyle changes and improved adherence to prescribed therapies. In attempting to understand these mechanisms, we have postulated a biphasic effect of cardiovascular therapies with initial treatment gains from driven by coronary revascularization and antiplatelet medications, with more gradual and persistent long-term benefits related to statin use (Table 1). It should be highlighted that the benefits of treatment arising from each of these therapies represents the *average* benefit seen across the respective trial cohorts. An important advantage of CCTA is the ability to target therapies at individuals according to their anatomically defined risk of events, a feature we identified within the SCOT-HEART trial (Fig. 1).

**Table 1:** Treatment effects observed in prospective trials of cardiovascular therapies according to clinical context.

Therapy	Clinical context	Risk ratio for myocardial infarction (95% confidence interval)	Selected references
Aspirin	Unstable angina	0.45 (0.35–0.58)	^31^^,^^32^
	Stable coronary disease	0.69 (0.60–0.80)	^33^
Statins	Primary prevention	0.46 (0.30–0.70)	^19^
	Stable coronary disease	0.69 (0.65–0.73)	^34^
Coronary revascularization	Acute coronary syndrome	0.79 (0.63–1.00)	^35^

Myocardial infarction is predominantly caused by acute coronary thrombosis secondary to plaque rupture or erosion. These events occur on non-obstructive coronary artery plaques. In both the SCOT-HEART and the Prospective Multicenter Imaging Study for Evaluation of Chest Pain (PROMISE) trials, most myocardial infarctions occurred in patients with non-obstructive coronary artery disease.^10^^,^^22^ Furthermore, over a half of myocardial infarctions occurred in patients with a normal functional stress test in the PROMISE trial.^6^ Thus, the prevention of myocardial infarction necessitates a technique that can identify non-obstructive coronary artery disease, and CCTA is the only current non-invasive technique that can achieve this. In the SCOT-HEART trial, whilst all subgroups benefited, the numerically largest relative risk reduction occurred in patients with non-anginal chest pain (0.45, 95% CI 0.19 to 1.03) and those diagnosed without angina due to coronary heart disease. This underpins a significant proportion of the benefit: the treatment of covert non-obstructive coronary artery disease.

## 2 Consistency of our findings

### 2.1 Internal consistency

We have previously reported the consistency of our findings across all major patient subgroups. This included the baseline characteristics used for minimization of treatment assignment including age, sex, established history of coronary heart disease, prior diagnosis of diabetes mellitus, and treating centre. In addition, there were no differences in treatment effect identified in relation to estimated 10-year cardiovascular risk or chest pain symptom typicality.^23^ Moreover, the effect size was very consistent with time, with similar proportionate reductions in events between our initial (1.7 years of follow up)^15^ and recent (4.8 years of follow-up)^13^ reports.

### 2.2 External consistency

Although the findings from the SCOT-HEART trial may appear contrasting to other studies, closer inspection demonstrates important commonalities. The CAPP^24^ (Cardiac CT for the Assessment of Pain and Plaque) and CRESCENT^25^ (Computed Tomography vs. Exercise Testing in Suspected Coronary Artery Disease) trials randomized 500 and 350 patients to CCTA respectively with approximately 1 year of follow-up in each case. Both trials showed increased diagnosis of coronary heart disease and consequently increased use of preventative medical therapies in the CCTA groups. In addition, despite being clearly underpowered for clinical events, both trials demonstrated numerically lower rates of myocardial infarction amongst those assigned to CCTA. However, perhaps a more appropriate comparator is the larger PROMISE trial which randomized more than 10,000 North American patients with suspected stable angina and reported a neutral outcome for the primary endpoint of death, myocardial infarction, hospitalization for unstable angina, or major procedural complication after 2 years of follow up. Compared with SCOT-HEART, the PROMISE trial cohort had less than half the prevalence of obstructive coronary disease identified on CCTA.^22^ In addition to this discrepancy in baseline risk, differences in trial design that may credibly account for the apparent contrasts in outcome have previously been described.^26^ One such difference relates to the primary endpoint selection. In the SCOT-HEART trial, we chose to focus on fatal and non-fatal myocardial infarction as the event most likely to benefit from coronary imaging with CCTA. In the PROMISE trial, there were directionally opposing effects of myocardial infarction and hospitalisation for unstable angina leading to an overall neutral effect. However, the PROMISE investigators did report that CCTA was associated with a 34% relative reduction in all-cause death and myocardial infarction at 12 months (hazard ratio 0.66 (95% confidence intervals, 0.44–1.00), P = 0.049). In addition to these individual studies, a meta-analysis of RCTs comparing CCTA with standard care, published in 2016 identified an incidence rate ratio for myocardial infarction of 0.69 (95% CI 0.49–0.98; p = 0.038),^27^ a result entirely consistent with the recent SCOT-HEART findings and confirmed in 2 subsequent larger meta-analyses by independent groups.^28^^,^^29^ Finally, reductions in myocardial infarction have also been reported in a very large (n = 86,705) observational Danish registry (HR for CCTA: 0.71, 95% CI 0.61 to 0.82).^30^

## 3 Conclusions

The SCOT-HEART trial has demonstrated the use of CCTA in addition to standard care to result in an important reduction in coronary heart disease death or non-fatal myocardial infarction. By merit of the large trial population and availability of long-term outcome data, this is the first time such a benefit has been definitively demonstrated. Our findings are internally consistent across treatment centres and patient subgroups, and are externally consistent with existing evidence from other randomised controlled trials and observational registries. The magnitude of the treatment effect appears large, but the confidence intervals remain overlapping with plausible beneficial effects of prescribed therapies and early coronary revascularization. Ultimately, the improved diagnosis and treatment of angina pectoris coupled together with the treatment of covert non-obstructive coronary artery disease underlies and explains the important beneficial effects of CCTA.
